# Tunnel Technique in Bone Augmentation Procedures for Dental Implant Rehabilitation: A Systematic Review

**DOI:** 10.3390/dj12120405

**Published:** 2024-12-11

**Authors:** Stefano Sivolella, Giulia Brunello, Dario Azeglio Castagna, Francesco Cavallin, Ugo Consolo

**Affiliations:** 1 Department of Neurosciences, Dentistry Section, University of Padova, 35128 Padova, Italy; stefano.sivolella@unipd.it (S.S.); darioazeglio.castagna@studenti.unipd.it (D.A.C.); 2 Department of Oral Surgery, University Hospital Düsseldorf, 40225 Düsseldorf, Germany; 3 Department of Orthodontics and Dentofacial Orthopedics, Corporate Member of Freie Universität Berlin and Humboldt-Universität zu Berlin, Charité–Universitätsmedizin Berlin, 14197 Berlin, Germany; 4 Independent Researcher, 36020 Solagna, Italy; cescocava@libero.it; 5 Surgical, Medical and Dental Department of Morphological Sciences related to Transplant, Oncology and Regenerative Medicine, University of Modena and Reggio Emilia, 41125 Modena, Italy; ugo.consolo@unimore.it

**Keywords:** alveolar ridge augmentation, bone regeneration, bone grafting, surgical flap

## Abstract

**Background/Objectives**: This systematic review aimed to compare the tunnel technique for pre-implant bone regeneration with traditional flap techniques also involving a crestal incision, in terms of procedure success, graft healing, postoperative course, patient satisfaction, and implant follow-up. **Methods**: A systematic search was conducted on MEDLINE/PubMed, EMBASE, and the Cochrane Central Register of Controlled Trials following PRISMA guidelines, searching for comparative prospective and retrospective studies in English, published between January 2002 and April 2024. The population of interest consisted of patients with edentulous ridge atrophy requiring pre-implant bone regeneration. The primary outcome was the success of the procedure. The secondary outcomes included complications, patient comfort, graft resorption, bone gain, primary implant stability, implant success/survival, peri-implant bone level change, and operative time. The risk of bias was assessed using RoB2 and ROBINS-I. **Results**: The search and selection process yielded one randomized controlled trial and three comparative observational studies, all with serious/high risk of bias. A narrative synthesis was conducted due to the small number of studies and the heterogeneity in key features. The tunnel technique might provide some advantages in terms of the success of the procedure, but the findings were not statistically significant. Conflicting findings or non-significant differences were reported in terms of the secondary outcomes. **Conclusions**: This review suggested some potential advantages of the tunnel technique for bone augmentation over traditional techniques involving a crestal incision, but the limited quality and amount of data precluded any definitive conclusions.

## 1. Introduction

Dental implant rehabilitation of severely resorbed edentulous alveolar ridges requires bone regeneration procedures of varying complexity. For this purpose, a wide variety of surgical techniques and materials have been described. Among these, the most common are sinus lift, bone block graft, and guided bone regeneration (GBR), which employs a membrane of variable material and nature to guide the formation of new bone tissue [1]. These techniques usually entail the design of a full-thickness triangular or trapezoidal flap, characterized by a crestal and one or two vertical releasing incisions. Furthermore, periosteal releasing incisions are usually performed to facilitate tension-free closure. Despite these expedients, the risk of failure and the incidence of complications remain not negligible [2]. A common adverse event is wound dehiscence, which can lead to bacterial colonization and site infection. This occurrence often requires the removal of the membrane, if used, and the debridement of the graft [3].

Minimally invasive techniques are being increasingly used in the attempt to improve procedure outcomes and reduce complication rates. Among these approaches, the tunnel flap can be considered for bone regeneration to reestablish an adequate bone volume for dental implant insertion [4]. Initially introduced in the last century for preliminary ridge augmentations for removable prostheses [5,6,7], it was proposed in the early 2000s in association with other materials and techniques [8] and subsequently applied to pre-implant reconstructive surgery, both for crestal regeneration and sinus lift [4,9,10,11,12]. The technique involves a single vertical, buccal incision, made mesially or distally to the defect, extending beyond the mucogingival junction. A full-thickness mucoperiosteal tunnel is elevated above the defect to accommodate materials and membranes of various nature, avoiding further vertical or crestal incisions. In this way the integrity of the periosteum is preserved, hence ensuring a tight closure and theoretically maintaining its ability to provide pluripotent stem cells, growth factors, and blood supply [13]. This conservative approach could also positively influence patient comfort, given the reduced number of intraoral incisions [14].

In a clinical study involving 142 patients [11], vertical bone augmentation was performed in the posterior maxilla according to the split bone block technique in combination with sinus floor elevation using a tunneling approach. Three months after the first surgery the planned number of implants could be inserted without any complications. In another prospective study comparing crestal versus tunnel incision technique for the reconstruction of alveolar defects with autogenous bone blocks, soft tissue dehiscence and graft failure were significantly lower in patients undergoing the tunnel technique [15].

The tunnel technique allows the maintenance of the integrity of the periosteum close to the bone graft, and this could contribute to an enhanced vascularization of the area [4]. The application of a tunnel flap has similarities with flapless implant surgery, resulting in a minimally invasive technique that improves blood supply compared with flapped surgery [16,17]. However, there is still contradictory evidence about the advantages of the flapless over the flapped approach and, as a consequence, about the key role of the preservation of the periosteum [18,19,20].

Other critical aspects of the tunnel technique consist in the relatively restricted access to the grafted area and, in cases of complex anatomy, difficulties in proper mucoperiosteal flap elevation, leading to incomplete flap detachment [9] and insufficient vision and management of the operating field.

Therefore, the rationale of this systematic review was to clarify whether the tunnel technique for bone augmentation may improve the success of the procedure compared to traditional techniques also involving a crestal incision and reduce the incidence of complications.

## 2. Materials and Methods

### 2.1. Study Design

**Figure 1:**
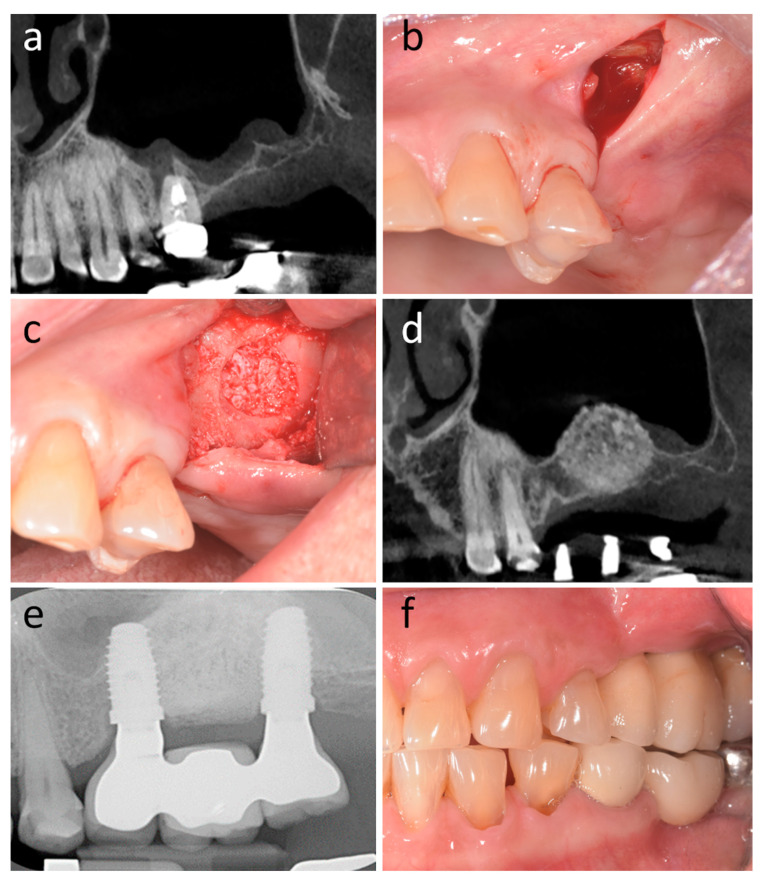
Representative clinical case of tunnel flap application belonging to the author’s (S.S.) original research data. (**a**) Preoperative CT demonstrating right upper alveolar process atrophy associated with edentulism. (**b**) Single buccal incision mesial to the antrostomy area. (**c**) Full-thickness flap elevated and heterologous bone graft in place at the end of the procedure. (**d**) Postoperative CT scan at 5 months with the graft in place. (**e**) Implants placed in the bone graft and prosthesis in place. (**f**) Intraoral view of the prosthesis on implants.

This is a systematic review of prospective and retrospective comparative studies about the tunnel technique in bone augmentation procedures for dental implant rehabilitation. A representative clinical case belonging to the author’s (S.S.) original research data is presented in Figure 1. The review was performed according to the Preferred Reporting Items for Systematic Reviews and Meta-Analyses (PRISMA) guidelines [21] and was registered in PROSPERO (CRD42023431359).

### 2.2. Search Strategy

We systematically searched MEDLINE/PubMed, EMBASE, and the Cochrane Central Register of Controlled Trials to detect eligible studies. In PubMed, the following search strategy was used: (tunnel or tunneling) and (oral or flap or “alveolar ridge” or “bone regeneration” or maxilla* or mandib*). The search strategy was customized to suit the other electronic sources (Supplementary Table S1). The lists from each source were combined and the duplicates were deleted. Two investigators (G.B., D.A.C.) independently assessed both titles and abstracts of the unique records and removed the studies outside the scope of the review. The full texts of all potentially eligible studies were retrieved and examined to exclude those not satisfying the inclusion criteria. Finally, we hand-searched the reference lists of the studies on tunnel technique for intraoral bone regeneration to identify additional studies of interest. Any disagreement was solved by consensus or with a third investigator (S.S.).

### 2.3. Criteria for Considering Studies for This Review

Condition: treatment of alveolar ridge defects using bone augmentation procedures.

Comparator: bone augmentation procedures and lateral sinus lift for intraoral implant rehabilitation using traditional flaps involving a crestal incision and vertical releasing incisions, when indicated, without subperiosteal tunnelization.

Intervention: tunnel technique for bone augmentation of atrophic alveolar ridges or sinus lift with lateral approach. The technique consists in elevating a full-thickness flap starting from a single vertical releasing incision, which is usually located medially to the grafted site, without the design of any crestal incision.

Outcomes: the primary outcome was the success of the bone augmentation procedure (defined as the possibility to insert implants as planned and/or no need for graft debridement during healing); the secondary outcomes included complications, patient comfort, graft resorption, histological assessment, primary implant stability, implant success/survival rate, peri-implant bone level change, and operative time.

Population: human subjects needing bone augmentation of edentulous alveolar ridges for dental implant rehabilitation.

Study design: prospective and retrospective comparative studies. Both randomized and non-randomized studies were included to gather a wider amount of information about the technique.

Time: between January 2002 and April 2024. The search was conducted starting from 2002 since the current application of the technique was described and introduced by Ylinen et al. [8].

Language: English.

### 2.4. Outcome Measures

The success of the bone augmentation procedure was defined as the possibility to insert implants as planned and/or no need for graft debridement during healing. The complications included graft exposure, temporary and permanent paresthesia, intra- and postoperative bleeding, swelling, and hematoma formation. Patient comfort was usually measured using a visual analogue scale (VAS-pain). The graft reabsorption was defined as the horizontal or vertical change in the profile of hard tissues during the healing of the surgical site, comparing immediate post-operative imaging to pre-implant imaging. The primary implant stability was defined as the stability of implants immediately after insertion. The implant survival was defined as the persistence of the fixture in the designated site, in the absence of indications for removal. The peri-implant bone level change was defined as the variation in distance between the most coronal margin of the implant collar and the most coronal point of the bone-implant contact. The operative time was defined as the time elapsed between incision and final suture. During data collection, we added the vertical and horizontal bone gain defined as the respective vertical or horizontal variation in bone profile between baseline and implant surgery intervention as a post-hoc outcome measure that could be interesting for the reader. Outcome measures also included histological assessment.

### 2.5. Data Collection

Two investigators (D.A.C., G.B.) independently extracted relevant data from included studies. Any inconsistency was solved by consensus or with a third investigator (S.S.). For each article, we extracted the following information: study design, sample size, number and experience of surgeons, demographics and health status of participants, site of surgical procedures, type of augmentation needed (vertical or horizontal, sinus lift), surgical technique for all groups, materials used for augmentation and technical variations, antibiotic prophylaxis (if any), follow-up timing and duration, complications, number of implants placed, timing of implant surgery (simultaneous or delayed), and data on the outcome measures.

### 2.6. Assessment of Risk of Bias

Two investigators (F.C., D.A.C.) independently evaluated the risk of bias of the included studies and any inconsistency was solved by consensus with all authors. The assessment was performed according to the criteria in the Cochrane Handbook for Systematic Reviews of Interventions [22]. The risk of bias was evaluated using the RoB2 (revised tool for Risk of Bias in randomized trials) tool for randomized controlled trials [23] and the ROBIN-I (Risk Of Bias in Non-randomized Studies of Interventions) tool for non-randomized controlled studies [24]. The risk of bias was categorized as serious/high, low, or unclear as described by the developers; if not available, the judgement was “unclear risk of bias”.

### 2.7. Data Synthesis

The procedure for screening and selection of the studies was displayed in a flow-chart. Relevant data were extracted from included studies and summarized in tables. The small number of the included studies and the heterogeneity in key features (such as study design, type of membrane, graft material, soft tissue expansion before procedure, and patient’s health status) precluded the feasibility of a meaningful meta-analysis, hence a narrative synthesis of the included studies was conducted. When appropriate, the synthesis included a measure of the effect (relative risk or mean difference, with 95% confidence interval) for each study separately. The agreement between investigators during study selection was assessed using Cohen’s kappa.

## 3. Results

Details on the adherence to the updated PRISMA guidelines are presented in Attachment 1 [21].

### 3.1. Search Results

**Figure 2:**
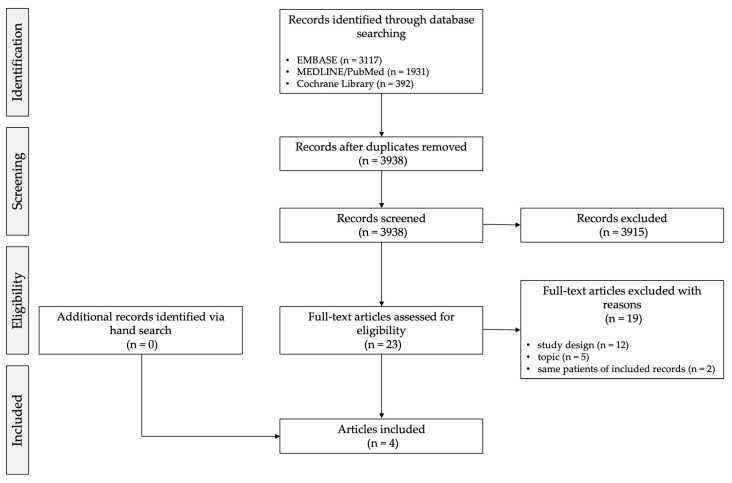
Flowchart of selection process.

The search identified 3938 non-duplicated records. After excluding 3915 records based on title/abstract, 23 potentially eligible records were retrieved for full-text review. Of these, 17 were excluded due to different design (n = 12) [4,25,26,27,28,29,30,31,32,33,34,35] or different topic (n = 5) [36,37,38,39,40]. Two additional records were excluded because they reported the same cases of other records [41,42]. The agreement between investigators was moderate during the title/abstract selection stage (Cohen’s kappa 0.51) and substantial during the full-text selection stage (Cohen’s kappa 0.70). No additional articles were identified via hand search. Finally, four articles were included in the data synthesis (Figure 2) [12,15,43,44].

### 3.2. Study and Patient Characteristics

The analysis included one randomized controlled trial [12], two controlled prospective studies [15,44] and one retrospective cohort study [43]. The characteristics of the included studies are summarized in Table 1. The sample size ranged from 30 to 68 patients. One study did not enroll smokers [12], while this exclusion criteria was not explicit in the other three studies. Information on the surgical procedures, such as vertical length of incision, or material for bone augmentation, is reported in Supplementary Tables S2–S5. Follow-up ranged from 6 to 24 months.

### 3.3. Risk of Bias in Included Studies

The risk of bias is summarized in Table 2. All observational studies were judged at serious risk of bias in at least one domain [15,43,44], including bias due to confounding (n = 3), bias due to deviations from intended interventions (n = 1) and bias in selection of the reported result (n = 1). In details, one study included retrospective cohorts, implying possible treatment preferences depending on the initial status of the patients [43]. Another study included consecutive surgeries alternating tunnel and crestal technique, thus introducing possible imbalances between the intervention groups, and offered unclear reporting of some outcomes measured, i.e., VAS and operative time [15]. The third observational study did not offer details about the patient characteristics, hence possible imbalances between the groups could not be excluded [44].

Finally, the randomized control trial was judged at overall high risk of bias due to high risk of bias arising from the randomization process and due to deviations from the intended interventions [12]. In addition, there were some concerns about missing outcome data and biased measurement of the outcome.

### 3.4. Narrative Synthesis on the Success of the Bone Augmentation Procedure

Two studies investigated the success of the bone augmentation procedure (Table 3). Deeb et al. [43] reported success in 18/21 (86%) patients with the tunnel technique and 22/31 (71%) patients with the crestal procedure (relative risk 1.20, 95% confidence interval 0.91 to 1.61). Altiparmak et al. [15] reported success in 37/38 (97%) sites with the tunnel technique and 34/37 (92%) sites with the crestal procedure (relative risk 1.06, 95% confidence interval 0.95 to 1.18).

### 3.5. Narrative Synthesis on the Complications

All studies investigated the exposure of the graft, which ranged within 0–19% with the tunnel technique and 9–52% with the crestal procedure (Table 3). The relative risk was 0.37 (95% confidence interval 0.14 to 0.95) in Deeb et al. [43], 0.32 (95% confidence interval 0.13 to 0.80) in Altiparmak et al. [15], 0.14 (95% confidence interval 0.01 to 2.55) in Wychowanski et al. [44], and 0.20 (95% confidence interval 0.01 to 3.95) in Byun et al. [12].

One study investigated paresthesia (Table 3) [15]. The authors found temporary paresthesia in 4/38 (11%) sites with the tunnel technique and 6/37 (16%) sites with the crestal procedure (relative risk 0.65, 95% confidence interval 0.20 to 2.11), while they did not find any permanent paresthesia.

None of the included studies reported intra- and postoperative bleeding, swelling, or hematoma formation.

### 3.6. Narrative Synthesis on Graft Reabsorption

One study investigated graft reabsorption (Table 3) [12]. The authors found a mean of 1.57 mm (standard deviation 1.03) with the tunnel technique and 2.32 mm (standard deviation 1.09) with the crestal procedure (mean difference −0.75, 95% confidence interval −1.38 to −0.12).

### 3.7. Narrative Synthesis on Histological Evaluation

None of the included studies reported on histological assessment.

### 3.8. Narrative Synthesis on the Primary Implant Stability

One study investigated the primary implant stability using the periotest value (PTV) (Table 3) [44]. The authors found a mean of −1.2 (standard deviation 1.6) with the tunnel technique and −3.2 (standard deviation 1.3) with the crestal procedure (mean difference 2.0, 95% confidence interval 0.9 to 3.1).

### 3.9. Narrative Synthesis on the Implant Survival

One study investigated implant survival (Table 3) [44]. With the tunnel technique 29/30 (97%) implants survived, while 26/30 (87%) survived with the crestal procedure (relative risk 0.25, 95% confidence interval 0.03 to 2.10).

### 3.10. Narrative Synthesis on the Peri-Implant Bone Level Change

Two studies investigated the peri-implant bone level change (Table 3). Wychowanski et al. [44] reported that bone stability did not exceed 1.2 mm at 2 years after the implantation. Byun et al. [12] reported a mean of 0.52 mm (standard deviation 0.21) with the tunnel technique and 0.41 mm (standard deviation 0.22) with the crestal procedure (mean difference 0.11, 95% confidence interval −0.02 to 0.23).

### 3.11. Narrative Synthesis on Operative Time

One study investigated operative time [15], and reported a median of 95 and 96 min for the tunnel technique and the crestal procedure, respectively (Table 3).

### 3.12. Narrative Synthesis on Vertical and Horizontal Bone Gain

Only two studies reported quantitative information on bone gain based on cone-beam computed tomography scans. Wychowanski et al. [44] reported a mean vertical bone gain of 4.4 mm (standard deviation 1.5) with the tunnel technique and 4.3 mm (standard deviation 1.3) with the crestal procedure (mean difference 0.10, 95% confidence interval −0.95 to 1.15) (Table 3). Byun et al. [12] reported a mean vertical bone gain of 3.55 and 1.90 mm for the tunnel technique and the crestal procedure, respectively, at 6 months after bone augmentation (Table 3). At the same time point, higher mean horizontal bone gain was observed in the tunnel group as compared to the crestal one.

### 3.13. Narrative Synthesis on Patient Comfort

One study investigated patient comfort immediately after surgery and at 1 month after surgery [15]. The authors used a visual analogue scale (VAS) ranging from 0 cm (no pain) to 10 cm (most severe pain) but did not report any values in their paper.

## 4. Discussion

This systematic review summarized available information on the comparison between tunnel technique for bone augmentation and traditional techniques involving a crestal incision. Overall, the tunnel technique might offer some advantages but the low number of relevant studies, the small sample size, and the large heterogeneity in key features precluded any definitive conclusions.

In general, the tunnel technique appeared safe and effective. The success rate was higher in patients treated with the tunnel technique, although the difference between tunnel and crestal techniques was not statistically different [15,43]. Graft exposure is a recurrent inconvenience of commonly used bone grafting techniques, associated with an increased risk of infection of the site and partial or total removal of the graft [2]. Among the included studies, graft exposure occurred more frequently with the crestal approach than with the tunnel technique, which showed values ranging between 0 and 19%. A low exposure rate has been reported with the tunnel technique also in a 10-year clinical study on mandibular crest augmentation using the tunnel technique, i.e., two graft exposures over 128 grafted sites (1.6%) [35]. Not only the adopted flap design but also the type of barrier could influence the exposure rate [45]. In a systematic review including 667 sites treated with titanium meshes [46], approximately one third of the cases presented mesh exposure, which, however, rarely compromised the success of the procedure.

Notably, Byun et al. used a tissue expander in combination with the tunnel technique [12]. The effects of this practice require further investigation, although pre-augmentation soft tissue expansion, even when combined with crestal incision techniques, has been suggested to decrease the risk of dehiscence by improving the vascularization of the soft tissues in the graft region and reducing flap tension [47].

No permanent paresthesia was registered, whereas temporary paraesthesia was reported in one study, with no statistically significant difference between tunnel and crestal techniques [15]. Moreover, no author accounted for additional complications such as intra- or postoperative bleeding, swelling or hematoma formation, which are important factors for patient comfort, nor for indirect indicators such as the number of painkillers taken. As regards pain, in one included study no statistically significant difference in intraoperative and postoperative VAS scores was detected between tunnel and crestal techniques for both the ramus or symphysis donor site [15]. Unfortunately, the authors did not report any numerical values that can inform the reader about patient satisfaction, which is one of the main goals of minimally invasive surgical techniques. Postoperative symptoms and oral health-related quality of life are significantly impacted by pain, swelling, difficulty of mouth opening, surgery duration, and flap advancement following guided bone regeneration [48]. Patient-reported outcome measures (PROMs) seem to be seldom investigated in oral surgery. The need for a more extensive use and standardization of PROMs has been highlighted by several authors [49,50]. A review of the efficacy of biologics for alveolar ridge preservation/reconstruction and implant site development reported that only four of the 39 included articles evaluated PROMs [51]. Similarly, in another systematic review focused on bone preservation or augmentation, PROMs were reported only in 15.7% of studies [50].

Of note, radiological examinations were reported in three studies [12,43,44], of which only two presented radiographic follow-up data of the placed implants. Furthermore, imaging analyses were often incomplete and generally insufficient to determine if the tunnel technique could be advantageous in terms of graft healing and implant success.

A negligible difference in vertical bone gain between tunnel and crestal techniques was reported in one included study [12]. In addition, the concomitant use of a tissue expander in the tunnel group might have affected the observed result [12]. Similarly, the lesser graft resorption during healing could not be definitively attributed to the tunnel technique.

Peri-implant bone level change was not statistically different between tunnel and crestal techniques [12], and it did not exceed 1.2 mm at 2 years after the implantation [44]. However, the different surgical protocols and graft materials in the two groups suggest caution in the interpretation of such findings. The experience of the operators may affect the outcome of the tunnel technique, which can be more complex than procedures involving a crestal incision. Unfortunately, we could not assess such aspects due to lack of clear information.

Similarly, the complexity of the tunnel technique may suggest a prolonged operative time. In our review of the literature, only one study reported such information, indicating basically the same duration (95–96 min) for both procedures [15]. Autologous bone blocks were used, and their harvesting, included in the time calculation, is known to be time-consuming [52,53]. This finding was in agreement with a previous study reporting a mean time of 87 min for regenerative procedures using the tunnel flap technique in conjunction with autologous bone blocks [10]. This outcome seems promising given the greater complexity of the technique and the increased stress for both patient [54] and operator, as well as higher chairside costs, which are associated with longer procedures. The large heterogeneity of the procedures in terms of mandibular and maxillary localization, biomaterials, and vertical or horizontal augmentation, coupled with the small sample sizes (Supplementary Tables S2, S3 and S5), did not allow for stratification and analysis of whether periosteum preservation and tension-free closure in the tunnel technique may be particularly beneficial in more challenging situations with high risk of dehiscence and failure. The main awaited features of the tunnel technique, as well as flapless oral surgeries (i.e., alveolar ridge preservation, flapless guided implant surgery), are the minimal invasiveness and the maintenance of the integrity of the periosteum [4,16,17]. In this regard, interesting findings were reported in the study of Khoury et al. [55], that did not meet the inclusion criteria of the present review. The authors tested the tunnel flap combined with various regenerative procedures on 15 selected patients undergoing bisphosphonate treatment for osteometabolic reasons, and they observed excellent results even in conditions that can be generally considered a contraindication for bone regeneration. During graft healing, only three sites exhibited incomplete integration, with soft tissue migration. This complication was addressed with an additional autologous bone graft performed at the time of implant surgery, and all 71 planned implants were inserted, 28 simultaneously and 43 after a healing period of three months. At three years, only one implant was lost due to lack of osseointegration [55].

The analysis of the risk of bias highlighted that all included studies were judged at serious/high risk of bias, suggesting some caution in the interpretation of their findings. In the observational studies [15,43,44], the main source of concerns was the possible imbalances between the treatment groups which might have influenced the results. In the randomized control trial [12], the main sources of concern involved research methodology and treatment strategy.

The literature on the tunnel technique offered other interesting studies which did not fulfill the inclusion criteria of this review but can provide additional information on the topic [9,10,11,14,55,56]. In a large clinical study [11], 154 regeneration procedures were performed in the posterior maxilla, combining the split bone block technique with maxillary sinus lifts, accessing the site via the tunnel flap. A 100% success rate was reported in terms of the ability to place implants according to the initial planning. Additionally, early graft dehiscence occurred in few smoking patients (3.9%) [11]. In another study, the tunnel technique was applied in 50 procedures on 43 patients using autologous corticocancellous bone blocks and cancellous particulate [10]. Among these, two cases resulted in failure with graft loss, while the eight cases of dehiscence were successfully resolved with graft osteoplasty. De Stavola and Tunkel [9] described the use of the tunnel technique with autologous corticocancellous bone blocks and autologous cancellous particulate, combining external bone augmentation with sinus lift when appropriate. Ten patients with mandibular or maxillary defects were treated. All sites healed without complications, and no dehiscence was observed. Furthermore, all implants could be placed as planned after graft healing. In another study, the treatment of 20 sites in 15 patients were reported with combined horizontal and vertical defects in the posterior mandibular region [56]. Bone blocks harvested from the coronoid process were positioned via a subperiosteal tunnel. One site showed dehiscence, effectively treated with graft osteoplasty and saline rinses for 15 days. Patient satisfaction was excellent, with one patient experiencing prolonged swelling and two reporting moderate pain. Graft resorption during healing was modest, with an average horizontal resorption of 16.88% and vertical resorption of 25.37% at the 6-month follow-up.

The findings of our review seem to confirm the validity of the tunnel technique for bone regeneration. On the other hand, the absence of a crestal incision and small access size may result in limited visibility [11], especially in the presence of complex anatomies [4]. In addition, proper filling of the defect may be difficult in some cases, especially in the anterior region of the tunnel [57], and management of potential complications may be less straightforward for a less trained operator. Finally, contextual implant placement may be difficult or impossible, limiting the applicability of the technique.

The present systematic review has some limitations that should be considered by the reader, first of all the large heterogeneity in key features of the included studies, differing in their designs, patient populations, surgical techniques, graft materials, and follow-up durations. This precluded the pooling of the results; hence, only a narrative synthesis of the findings was feasible, and no meta-analysis could be performed. Furthermore, the available data did not allow drawing strong conclusions on the outcomes of interest because some were investigated by only one study. The small sample size of the included studies suggests caution in the interpretation of their findings. Finally, the high risk of bias in the included studies represents a critical weakness of the present review. Indeed, all four studies were found to have serious or high risk of bias in key domains, including confounding and deviations from intended interventions. As a result, the reliability of the reported outcomes necessitates cautious interpretation.

Overall, the present systematic review suggests that high quality studies are needed to provide reliable information on the topic. Randomized clinical trials comparing tunnel versus other kind of flap designs for alveolar bone augmentation should be conducted comprising at least a core set of recommended outcomes for clinical trials in implant dentistry and bone augmentation, including histological investigations and adequate imaging assessments [50,58]. Besides clinician-reported outcome measures (CROMs), patient-reported outcome measures (PROMs) should be taken into account.

## 5. Conclusions

The literature suggested some potential advantages of the tunnel technique for bone augmentation over traditional techniques involving a crestal incision, but the limited quality and amount of data precluded any definitive conclusions. Large, high-quality studies are needed to provide reliable information on the topic. Further investigations may benefit from the definition of some standardization in the study design to prevent heterogeneity in key features (such as type of membrane, graft material, soft tissue expansion, and patient’s health status) and improve comparability and generalizability of the findings. In addition, future studies may also investigate patient comfort and the learning curve of the surgeons with the tunnel technique and employ a more comprehensive radiological evaluation.

## Figures and Tables

**Table 1:** Characteristics of included studies.

Author(s), Year	Study Design	Males: Females	Age, Mean	Health Status	Smokers, n	Patients (Cases: Controls), n	Sites (Cases: Controls), n	Surgeons, n	Surgeon Experience	Follow-Up Duration
Deeb et al., 2016 [43]	Retrospective cohort study	NR	>18 years	NR	NR	52 (21:31)	52 (21:31)	Multiple (unspecified number)	Senior level oral and maxillofacial surgery residents	6 months
Altiparmak et al., 2017 [15]	Controlled prospective study	29:39	41.5 years	ASA I (healthy subjects)	NR	68 (NR)	75 (38:37)	One	NR	6 months
Wychowanski et al., 2020 [44]	Controlled prospective study	NR	NR	Immunocompromised subjects	NR	30 (15:15)	30 (15:15)	NR	NR	24 months
Byun et al., 2020 [12]	Randomized controlled trial	24:22	57.6 years	Among exclusion criteria: metabolic diseases, hemorrhagic diseases, specific therapies (e.g., drugs affecting bone metabolism)	None	46 (23:23)	46 (23:23)	Multiple (unspecified number)	NR	7–19 months

ASA: American Society of Anesthesiologists; NR: not reported.

**Table 2:** Assessment of the risk of bias of included studies.

Author(s), Year	Study Design	Tool	Domain	Risk of Bias	Overall Risk of Bias
Deeb et al., 2016 [43]	Retrospective cohort study	ROBINS-I	Bias due to confounding	Serious	Serious
			Bias in selection of participants	Low	
			Bias in classification of interventions	Low	
			Bias due to deviations from intended interventions	Low	
			Bias due to missing data	Low	
			Bias in measurement of outcomes	Low	
			Bias in selection of the reported result	Low	
Altiparmak et al., 2017 [15]	Controlled prospective study	ROBINS-I	Bias due to confounding	Serious	Serious
			Bias in selection of participants	Low	
			Bias in classification of interventions	Low	
			Bias due to deviations from intended interventions	Low	
			Bias due to missing data	Low	
			Bias in measurement of outcomes	Low	
			Bias in selection of the reported result	Serious	
Wychowanski et al., 2020 [44]	Controlled prospective study	ROBINS-I	Bias due to confounding	Serious	Serious
			Bias in selection of participants	Low	
			Bias in classification of interventions	Low	
			Bias due to deviations from intended interventions	Serious	
			Bias due to missing data	Unclear	
			Bias in measurement of outcomes	Low	
			Bias in selection of the reported result	Low	
Byun et al., 2020 [12]	Randomized controlled trial	RoB2	Risk of bias arising from the randomization process	High	High
			Risk of bias due to deviations from the intended interventions	High	
			Risk of bias due to missing outcome data	Some concerns	
			Risk of bias in measurement of the outcome	Some concerns	
			Risk of bias in selection of the reported result	Low	

**Table 3:** Summary of findings.

Author(s), Year	Success of the Bone Augmentation Procedure, n/N (%)		Exposure of the Graft, n/N (%)		Temporary Paresthesia, n/N (%)		Permanent Paresthesia, n/N (%)		Graft Resorption, Mean (SD)		Primary Implant Stability, Mean (SD)		Implant Success/Survival rate, n/N (%)		Peri-Implant Bone Level Change, Mean (SD)		Operative Time, Median		Vertical Bone Gain, Mean (SD)	
	**T**	**C**	**T**	**C**	**T**	**C**	**T**	**C**	**T**	**C**	**T**	**C**	**T**	**C**	**T**	**C**	**T**	**C**	**T**	**C**
Deeb et al., 2016 [43]	18/21 (86%)	22/31 (71%)	4/21 (19%)	16/31 (52%)	NR	NR	NR	NR	NR	NR	NR	NR	NR	NR	NR	NR	NR	NR	NR	NR
Altiparmak et al., 2017 [15]	37/38 (97%)	34/37 (92%)	5/38 (13%)	15/37 (41%)	4/38 (11%)	6/37 (16%)	Nil	Nil	NR	NR	NR	NR	NR	NR	NR	NR	95 min	96 min	NR	NR
Wychowanski et al., 2020 [44]	NR	NR	0/15 (0%)	3/15 (20%)	NR	NR	NR	NR	NR	NR	−1.2 (1.6)	−3.2 (1.3)	29/30 (97%)	26/30 (87%)	<1.2 mm	<1.2 mm	NR	NR	4.4 mm (1.5)	4.3 mm (1.3)
Byun et al., 2020 [12]	NR	NR	0/23 (0%)	2/23 (9%)	NR	NR	NR	NR	1.57 mm (1.03)	2.32 mm (1.09)	NR	NR	NR	NR	0.52 mm (0.21)	0.41 mm (0.22)	NR	NR	3.55 mm (NR)	1.9 mm (NR)

C: crestal; NR: not reported; SD: standard deviation; T: tunnel.

## Data Availability

Data are available from the corresponding author upon reasonable request.

## Supplementary Materials

The following supporting information can be downloaded at: https://www.mdpi.com/article/10.3390/dj12120405/s1, Table S1: Search strategies for the different databases; Table S2: Characteristics of the tunnel procedure in the included studies; Table S3: Characteristics of the crestal procedure in the included studies; Table S4: Other information on procedures in the included studies; Table S5: Other information on location and type of atrophy; Attachment 1: PRISMA 2020 checklist.

## References

[1] Tolstunov L., Hamrick J.F.E., Broumand V., Shilo D., Rachmiel A. (2019). Bone Augmentation Techniques for Horizontal and Vertical Alveolar Ridge Deficiency in Oral Implantology. Oral Maxillofac. Surg. Clin..

[2] Sanz-Sánchez I., Sanz-Martín I., Ortiz-Vigón A., Molina A., Sanz M. (2022). Complications in bone-grafting procedures: Classification and management. Periodontol. 2000.

[3] Garcia J., Dodge A., Luepke P., Wang H.L., Kapila Y., Lin G.H. (2018). Effect of membrane exposure on guided bone regeneration: A systematic review and meta-analysis. Clin. Oral Implants Res..

[4] Karmon B., Tavelli L., Rasperini G. (2020). Tunnel Technique with a Subperiosteal Bag for Horizontal Ridge Augmentation. Int. J. Periodontics Restor. Dent..

[5] Rothstein S.S., Paris D.A., Zacek M.P. (1984). Use of hydroxylapatite for the augmentation of deficient alveolar ridges. J. Oral Maxillofac. Surg..

[6] Vanassche B.J., Stoelinga P.J., de Koomen H.A., Blijdorp P.A., Schoenaers J.H. (1988). Reconstruction of the severely resorbed mandible with interposed bone grafts and hydroxylapatite. A 2–3 year follow-up. Int. J. Oral Maxillofac. Surg..

[7] Kent J.N., Finger I.M., Quinn J.H., Guerra L.R. (1986). Hydroxylapatite alveolar ridge reconstruction: Clinical experiences, complications, and technical modifications. J. Oral Maxillofac. Surg..

[8] Ylinen P., Suuronen R., Taurio R., Törmälä P., Rokkanen P. (2002). Use of hydroxylapatite/ polymer-composite in facial bone augmentation. An experimental study. Int. J. Oral Maxillofac. Surg..

[9] De Stavola L., Tunkel J. (2013). Results of vertical bone augmentation with autogenous bone block grafts and the tunnel technique: A clinical prospective study of 10 consecutively treated patients. Int. J. Periodontics Restor. Dent..

[10] Restoy-Lozano A., Dominguez-Mompell J.L., Infante-Cossio P., Lara-Chao J., Espin-Galvez F., Lopez-Pizarro V. (2015). Reconstruction of mandibular vertical defects for dental implants with autogenous bone block grafts using a tunnel approach: Clinical study of 50 cases. Int. J. Oral Maxillofac. Surg..

[11] Khoury F., Hanser T. (2019). Three-Dimensional Vertical Alveolar Ridge Augmentation in the Posterior Maxilla: A 10-year Clinical Study. Int. J. Oral Maxillofac. Implants.

[12] Byun S.H., Kim S.Y., Lee H., Lim H.K., Kim J.W., Lee U.L., Lee J.B., Park S.H., Kim S.J., Song J.D. (2020). Soft tissue expander for vertically atrophied alveolar ridges: Prospective, multicenter, randomized controlled trial. Clin. Oral Implants Res..

[13] Lin Z., Fateh A., Salem D.M., Intini G. (2014). Periosteum: Biology and applications in craniofacial bone regeneration. J. Dent. Res..

[14] Kim H.S., Kim Y.K., Yun P.Y. (2016). Minimal invasive horizontal ridge augmentation using subperiosteal tunneling technique. Maxillofac. Plast. Reconstr. Surg..

[15] Altiparmak N., Uckan S., Bayram B., Soydan S. (2017). Comparison of Tunnel and Crestal Incision Techniques in Reconstruction of Localized Alveolar Defects. Int. J. Oral Maxillofac. Implants.

[16] Llamas-Monteagudo O., Girbés-Ballester P., Viña-Almunia J., Peñarrocha-Oltra D., Peñarrocha-Diago M. (2017). Clinical parameters of implants placed in healed sites using flapped and flapless techniques: A systematic review. Med. Oral Patol. Oral Cir. Bucal.

[17] Kim J.I., Choi B.H., Li J., Xuan F., Jeong S.M. (2009). Blood vessels of the peri-implant mucosa: A comparison between flap and flapless procedures. Oral Surg. Oral Med. Oral Pathol. Oral Radiol. Endodontol..

[18] Siu T.L., Dukka H., Saleh M.H.A., Tattan M., Dib Z., Ravidà A., Greenwell H., Wang H.L., Araujo M.G. (2023). Flap versus flapless alveolar ridge preservation: A clinical and histological single-blinded, randomized controlled trial. J. Periodontol..

[19] Atieh M.A., Alfardan L., Alsabeeha N.H.M. (2022). Flapped versus flapless alveolar ridge preservation: A systematic review and meta-analysis. Int. J. Oral Maxillofac. Surg..

[20] Majid O.W. (2024). Does flapless immediate implant placement lead to significant preservation of buccal bone compared to flap surgical protocol?. Evid. Based Dent..

[21] Page M.J., McKenzie J.E., Bossuyt P.M., Boutron I., Hoffmann T.C., Mulrow C.D., Shamseer L., Tetzlaff J.M., Akl E.A., Brennan S.E. (2021). The PRISMA 2020 statement: An updated guideline for reporting systematic reviews. Bmj.

[22] Higgins J.P.T.T., Thomas J., Chandler J., Cumpston M., Li T., Page M.J., Welch V.A. (2022). Cochrane Handbook for Systematic Reviews of Interventions Version 6.3.

[23] Sterne J.A.C., Savović J., Page M.J., Elbers R.G., Blencowe N.S., Boutron I., Cates C.J., Cheng H.Y., Corbett M.S., Eldridge S.M. (2019). RoB 2: A revised tool for assessing risk of bias in randomised trials. Bmj.

[24] Sterne J.A., Hernán M.A., Reeves B.C., Savović J., Berkman N.D., Viswanathan M., Henry D., Altman D.G., Ansari M.T., Boutron I. (2016). ROBINS-I: A tool for assessing risk of bias in non-randomised studies of interventions. Bmj.

[25] Lee E.A., Prasad H., Lynch S. (2024). Sequential Human Histology Results of the Subperiosteal Minimally Invasive Aesthetic Ridge Augmentation Technique (SMART): A Chronologic Wound Healing Proof-of-Principle Study. Int. J. Periodontics Restor. Dent..

[26] Charavet C., Lecloux G., Vandenberghe B., Lambert F. (2021). Buccal bone regeneration combined with piezocision in adult orthodontic patients: Clinical, 3D radiographic, and patient-reported outcomes. J. Stomatol. Oral Maxillofac. Surg..

[27] Nevins M.L., Camelo M., Nevins M., Schupbach P., Friedland B., Camelo J.M., Kim D.M. (2009). Minimally invasive alveolar ridge augmentation procedure (tunneling technique) using rhPDGF-BB in combination with three matrices: A case series. Int. J. Periodontics Restor. Dent..

[28] Kfir E., Kfir V., Eliav E., Kaluski E. (2007). Minimally invasive guided bone regeneration. J. Oral Implantol..

[29] Angelo T., Marcel W., Andreas K., Izabela S. (2015). Biomechanical Stability of Dental Implants in Augmented Maxillary Sites: Results of a Randomized Clinical Study with Four Different Biomaterials and PRF and a Biological View on Guided Bone Regeneration. Biomed. Res. Int..

[30] Soltan M., Smiler D., Soltan C., Prasad H.S., Rohrer M.D. (2010). Bone grafting by means of a tunnel dissection: Predictable results using stem cells and matrix. Implant Dent..

[31] Akhil K.P., Pramashivaiah R., Prabhuji M.L.V., Tasleem R., Almubarak H., Bahamdan G.K., Luke A.M., Shetty K.P., Snigdha N.T., Bhavikatti S.K. (2023). Alveolar Ridge Augmentation Assessment Using a Minimalistic Approach, with and without Low-Level Laser Therapy (LLLT)—A Comparative Clinical Trial. Medicina.

[32] Johnson T.M., Baron D. (2018). Tunnel Access for Guided Bone Regeneration in the Maxillary Anterior. Clin. Adv. Periodontics.

[33] Smiler D., Soltan M., Lee J.W. (2007). A histomorphogenic analysis of bone grafts augmented with adult stem cells. Implant Dent..

[34] Heller A.L., Heller R.L. (2010). Tissue management protocol: “tunnel bone graft” technique. Dent. Today.

[35] Khoury F., Hanser T. (2022). 3D vertical alveolar crest augmentation in the posterior mandible using the tunnel technique: A 10-year clinical study. Int. J. Oral Implantol..

[36] Migliorati M., Amorfini L., Signori A., Biavati A.S., Benedicenti S. (2015). Clinical and Aesthetic Outcome with Post-Extractive Implants with or without Soft Tissue Augmentation: A 2-Year Randomized Clinical Trial. Clin. Implant Dent. Relat. Res..

[37] Papace C., Büsch C., Ristow O., Keweloh M., Hoffmann J., Mertens C. (2021). The effect of different soft-tissue management techniques for alveolar ridge preservation: A randomized controlled clinical trial. Int. J. Implant Dent..

[38] AlGhamdi A.S. (2013). Post-surgical complications of symphyseal block graft with and without soft tissue grafting. Saudi Med. J..

[39] Mahn D.H., Woodside J. (2012). Multidisciplinary rehabilitation: Tunnel grafting techniques. Dent. Today.

[40] Elaskary A.T., Gaweesh Y.Y., Maebed M.A., Cho S.-C., El Tantawi M. (2020). A Novel Method for Immediate Implant Placement in Defective Fresh Extraction Sites. Int. J. Oral Maxillofac. Implants.

[41] Deeb G.R., Tran D., Carrico C.K., Block E., Laskin D.M., Deeb J.G. (2017). How Effective Is the Tent Screw Pole Technique Compared to Other Forms of Horizontal Ridge Augmentation?. J. Oral Maxillofac. Surg..

[42] Byun S.H., Kim S.H., Cho S., Lee H., Lim H.K., Kim J.W., Lee U.L., Song W., Kim S.J., Kim M.K. (2020). Tissue Expansion Improves the Outcome and Predictability for Alveolar Bone Augmentation: Prospective, Multicenter, Randomized Controlled Trial. J. Clin. Med..

[43] Deeb G.R., Wilson G.H., Carrico C.K., Zafar U., Laskin D.M., Deeb J.G. (2016). Is the Tunnel Technique More Effective Than Open Augmentation with a Titanium-Reinforced Polytetrafluoroethylene Membrane for Horizontal Ridge Augmentation?. J. Oral Maxillofac. Surg..

[44] Wychowanski P., Woliński J., Morawiec T., Kownacki P., Starzynska A., Kosieradzki M., Fiedor P. (2020). Preliminary Clinical Data and the Comparison of the Safety and Efficacy of Autogenous Bone Grafts Versus Xenograft Implantations in Vertical Bone Deficiencies Before Dental Implant Installation. Transplant. Proc..

[45] Thoma D.S., Bienz S.P., Figuero E., Jung R.E., Sanz-Martín I. (2019). Efficacy of lateral bone augmentation performed simultaneously with dental implant placement: A systematic review and meta-analysis. J. Clin. Periodontol..

[46] Cunha G., Carvalho P.H.A., Quirino L.C., Torres L.H.S., Filho V.A.P., Gabrielli M.F.R., Gabrielli M.A.C. (2022). Titanium Mesh Exposure After Bone Grafting: Treatment Approaches—A Systematic Review. Craniomaxillofacial Trauma Reconstr..

[47] Asa’Ad F., Rasperini G., Pagni G., Rios H.F., Giannì A.B. (2016). Pre-augmentation soft tissue expansion: An overview. Clin. Oral Implants Res..

[48] Kofina V., Monfaredzadeh M., Rawal S.Y., Dentino A.R., Singh M., Tatakis D.N. (2023). Patient-reported outcomes following guided bone regeneration: Correlation with clinical parameters. J. Dent..

[49] Gotfredsen K. (2023). Patient-reported outcomes for bone regenerative procedures. Periodontol. 2000.

[50] Shi J.Y., Montero E., Wu X.Y., Palombo D., Wei S.M., Sanz-Sánchez I. (2023). Bone preservation or augmentation simultaneous with or prior to dental implant placement: A systematic review of outcomes and outcome measures used in clinical trials in the last 10 years. Clin. Oral Implant. Res..

[51] Suárez-López Del Amo F., Monje A. (2022). Efficacy of biologics for alveolar ridge preservation/reconstruction and implant site development: An American Academy of Periodontology best evidence systematic review. J. Periodontol..

[52] McAllister B.S., Haghighat K. (2007). Bone augmentation techniques. J. Periodontol..

[53] Esposito M., Grusovin M.G., Kwan S., Worthington H.V., Coulthard P. (2008). Interventions for replacing missing teeth: Bone augmentation techniques for dental implant treatment. Cochrane Database of Systematic Reviews.

[54] Liu X., Zhou Y. (2024). Risk factors of perioperative hypertension in dental implant surgeries with bone augmentation. Beijing Da Xue Xue Bao Yi Xue Ban.

[55] Khoury F., Hidajat H. (2016). Extensive Autogenous Bone Augmentation and Implantation in Patients Under Bisphosphonate Treatment: A 15-Case Series. Int. J. Periodontics Restor. Dent..

[56] De Riu G., Meloni M.S., Pisano M., Baj A., Tullio A. (2012). Mandibular coronoid process grafting for alveolar ridge defects. Oral Surg. Oral Med. Oral Pathol. Oral Radiol..

[57] Hasson O. (2007). Augmentation of deficient lateral alveolar ridge using the subperiosteal tunneling dissection approach. Oral Surg. Oral Med. Oral Pathol. Oral Radiol. Endodontology.

[58] Tonetti M.S., Sanz M., Avila-Ortiz G., Berglundh T., Cairo F., Derks J., Figuero E., Graziani F., Guerra F., Heitz-Mayfield L. (2023). Relevant domains, core outcome sets and measurements for implant dentistry clinical trials: The Implant Dentistry Core Outcome Set and Measurement (ID-COSM) international consensus report. J. Clin. Periodontol..

