# Supplementary material for: Tunnel Technique in Bone Augmentation Procedures for Dental Implant Rehabilitation: A Systematic Review
Source: Dent J (Basel). 2024 Dec 11;12(12):405. doi: 10.3390/dj12120405 (PMC11674451; doi:10.3390/dj12120405)
Supplement: Supplementary file 1 [file dentistry-12-00405-s001.zip › dentistry-3341327-supplementary materials.pdf]

Table S1. Search strategies for the different databases.

Database	Search Strategy
PubMed	(("tunnel"[All Fields] OR "tunneled"[All Fields] OR "tunneling"[All Fields] OR "tunnelings"[All Fields] OR "tunnelization"[All Fields] OR "tunnelized"[All Fields] OR "tunnelled"[All Fields] OR "tunnelling"[All Fields] OR "tunnels"[All Fields] OR ("tunnel"[All Fields] OR "tunneled"[All Fields] OR "tunneling"[All Fields] OR "tunnelings"[All Fields] OR "tunnelization"[All Fields] OR "tunnelized"[All Fields] OR "tunnelled"[All Fields] OR "tunnelling"[All Fields] OR "tunnels"[All Fields])) AND ("mouth"[MeSH Terms] OR "mouth"[All Fields] OR "oral"[All Fields] OR ("surgical flaps"[MeSH Terms] OR ("surgical"[All Fields] AND "flaps"[All Fields]) OR "surgical flaps"[All Fields] OR "flap"[All Fields]) OR "alveolar ridge"[All Fields] OR "bone regeneration"[All Fields] OR "maxilla*"[All Fields] OR "mandib*"[All Fields])) AND (2002:2024[pdat])
Embase via Ovid	((Tunnel or tunneling) and (oral or flap or "alveolar ridge" or "bone regeneration" or maxilla* or mandib*)).mp. [mp=title, abstract, heading word, drug trade name, original title, device manufacturer, drug manufacturer, device trade name, keyword heading word, floating subheading word, candidate term word] limit 1 to yr="2002 -Current"
Cochrane Central Register of Controlled Trials (CENTRAL)	Trials matching (Tunnel or tunneling) and (oral or flap or "alveolar ridge" or "bone regeneration" or maxilla* or mandib*) in Title Abstract Keyword - with Cochrane Library publication date Between Jan 2002 and April 2024 (Word variations have been searched)

Table S2. Characteristics of the tunnel procedure in the included studies.

Author(s), year	Tunnel procedure	Vertical length of incision	Distance of vertical incision from defect	Material for bone augmentation	Titanium screws (number, type, purpose)	Membrane	Suture (type/diameter)
Deeb et al., 2016	Single vertical incision approximately 1 cm distally to the graft site, starting just inferior to the mucogingival margin. Periosteal elevation in the area to be augmented. Bone placed by modified tuberculine syringe.	NR	10 mm	1:1 mix of mineralized freeze-dried bone allograft (Puros®, Zimmer Dental) and particulate bovine-derived hydroxyapatite (Bio-Oss®, Geistlich Pharma)	No	Unclear	NR
Altiparmak et al., 2017	Single vertical incision 5 mm mesially to the defect	NR	5 mm	• Bone block harvested with piezo from ramus or symphisis<br>• Bio-Oss® (Geistlich Pharma; 0.25-1.00 mm granules) to fill the gaps	2 titanium screws (length: 8 or 10 mm; diameter: 1.3 or 1.5 mm) to stabilize bone block, placed by dissecting the tunnel	Platlet-rich fibrin membrane	Vycril™ 3-0 (Ethicon)
Wychowanski et al., 2020	The authors refer to "Tunnel" without further description	NR	NR	Xenograft (unclear)	NR	NR	NR
Byun et al., 2020	Two incisions distally and mesially to bone defect to remove tissue expander. Subsequent bone grafting through the same incisions without full flap reflection.	5-10 mm	NR	Xeno-bone graft material (Bio-Oss®; Geistlich Pharma)	2 titanium screws to retain the expansor	Collagen membrane (Cytoplast® RTM collagen membrane; Osteogenics; OssMem®; Osstem)	Dermalon® monofilament nylon 4–0 or 5–0 (Covidien)

Table S3. Characteristics of the crestal procedure in the included studies.

Author(s), year	Crestal procedure	Distance of releasing incisions from defect	Material for bone augmentation	Titanium screws (y/n, number, aim)	Membrane (y/n, type)	Suture (type/diameter)
Deeb et al., 2016	Crestal incision bisecting the attached mucosa. Buccal and lingual full-thickness flaps were reflected.	NR	1:1 mix of mineralized freeze-dried bone allograft (Puros®, Zimmer Dental) and particulate bovine-derived hydroxyapatite (Bio-Oss®, Geistlich Pharma)	At least 1 titanium screw lingual/palatal, 2 buccal screws at least	Titanium-reinforced PTFE membrane	NR
Altiparmak et al., 2020	Crestal incision on the top of the crest and relaxing vertical incisions. Horizontal periosteal incisions parallel to the crestal incision for tension-free closure.	NR	• Bone block harvested with piezo from ramus or symphisis.<br>• Bio-Oss® (Geistlich Pharma; 0.25-1.00 mm granules) to fill the gaps	2 titanium screws to stabilize bone block	Platlet-rich fibrin membrane	Vycril™ 3-0 (Ethicon)
Wychowanski et al., 2020	NR	NR	Autogengenous ring shape grafts from mental region	NR	NR	NR
Byun et al., 2020	GBR with periosteal-releasing incisions. Mid-crestal and two divergent vertical incisions at least 5 mm away from the surgical site were made, elevating a full-thickness mucoperiosteal flap.	5 mm	Unclear	In case of insufficient graft stability, tenting screws were applied. The membrane was fixed on the buccal side using metal pins.	d-PTFE titanium-reinforced membrane (Cytoplast® Ti250XL; Osteogenics)	Multiple interrupted and horizontal mattress with Dermalon® monofilament nylon 4–0 or 5–0 (Covidien)

NR: not reported; PTFE: polytetrafluoroethylene

Table S4. Other information on procedures in the included studies.

Author(s), year	Antibiotics (y/n, type, posology)	Analgesics/ mouthwash etc.	Implants type	N. implants (planned/inserted) (case)	Implant protocol (case)	N. implants (planned/inserted) (control)	Implant protocol (control)
Deeb et al., 2016	2 g amoxicillin orally or 900 mg clindamycin intravenously preoperatively. 500 mg amoxicillin every 8 hours or 300 mg clindamycin every 6 hours for 1 week post-op	NR	NR	18 (86%)	Delayed insertion (6 mths post augmentation). Single-stage surgery if appropriate torque. Implant loading after 3 mths	22 (71%)	Delayed insertion (6 mths post augmentation). Removal of membrane and screws, single stage if appropriate torques
Altiparmak et al., 2017	NR	NR	Straumann® or Nobel Biocare	Unclear (59 placed)	Delayed insertion (6 mths post augmentation)	Unclear (63 placed)	Delayed insertion (6 mths post augmentation)
Wychowanski et al., 2020	NR	NR	Implants length: 8-11.5 mm; diameter: 3.5-4.5 mm	30 (2 per patient); all inserted	Delayed insertion (6 mths post tunnel), submerged healing, implant loading after 6 mths	30 (2 per patient); all inserted	Simultaneous implant placement, submerged healing, loading at 6 mths
Byun et al., 2020	Antibiotics administration without further explanation	Chlorhexidine gargles (0.2%)	Bone level SLActive® (Straumann®) and TS III SA® (Osstem)	Implant placement in all patients	NR	Implant placement in all patients	NR

Mths: months; NR: not reported

Table S5. Other information on location and type of atrophy.

Author(s), year	Type of augmentation technique	Type of edentulism	Type of atrophy
Deeb et al., 2016	Test: tunnel technique for crestal regeneration associated with particulate graft material<br>Control: guided bone regeneration	NR	Horizontal atrophy
Altiparmak et al., 2017	Test: tunnel technique for crestal augmentation associated with bone block<br>Control: bone block technique for crestal regeneration	Maxillary or mandibular edentulism	Vertical and horizontal defects. The mean size of grafts was 4 mm in width, 10 to 12 mm in length, and 6 mm in height
Wychowanski et al., 2020	Test: tunnel technique for crestal regeneration associated with particulate graft material<br>Control: bone block technique for crestal augmentation	Loss of premolar or molar teeth in mandible	Vertical atrophy
Byun et al., 2020	Test: tunnel technique for crestal regeneration associated with particulate graft material<br>Control: guided bone regeneration	Loss of one or more maxillary or mandibular teeth	Severe vertical atrophy
